# An Unmatched Radio Frequency Chain for Low-Field Magnetic Resonance Imaging

**DOI:** 10.3389/fphy.2021.727536

**Published:** 2021

**Authors:** Joshua R. Harper, Cristhian Zárate, Federico Krauch, Ivan Muhumuza, Jorge Molina, Johnes Obungoloch, Steven J. Schiff

**Affiliations:** 1 Center for Neural Engineering, Department of Engineering Science and Mechanics, The Pennsylvania State University, University Park, PA, United States; 2 Laboratory of Mechanics and Energy, Department of Engineering, National University of Asunción, Asunción, Paraguay; 3 Low Field MRI Lab, Department of Biomedical Engineering, Mbarara University of Science and Technology, Mbarara, Uganda; 4 Department of Neurosurgery, The Pennsylvania State University, University Park, PA, United States; 5 Department of Physics, The Pennsylvania State University, University Park, PA, United States

**Keywords:** low field MRI, low cost, low power, prepolarization MRI, low- to middle-income countries, radiofrequency, sustainable MRI, portable

## Abstract

Magnetic Resonance Imaging (MRI) is a safe and versatile diagnostic tool for intracranial imaging, however it is also one of the most expensive and specialized making it scarce in low- to middle-income countries (LMIC). The affordability and portability of low-field MRI offers the potential for increased access to brain imaging for diseases like Hydrocephalus in LMIC. In this tutorial style work, we show the design of a low powered and low cost radio frequency chain of electronics to be paired with a previously reported prepolarized low-field MRI for childhood hydrocephalus imaging in sub-Saharan Africa where the incidence of this condition is high. Since the Larmor frequency for this system is as low as 180 kHz, we are able to minimize the impedance of the transmit coil to 5 ohms rather than match to 50 ohms as is traditionally the case. This reduces transmit power consumption by a factor of 10. We also show the use of inexpensive and commonly available animal enclosure fencing (“chicken wire”) as a shield material at this frequency and compare to more traditional shield designs. These preliminary results show that highly portable and affordable low-field MRI systems could provide image resolution and signal-to-noise sufficient for planning hydrocephalus treatment in areas of the world with substantial resource limitations. Employment of these technologies in sub-Saharan Africa offers a cost-effective, sustainable approach to neurological diagnosis and treatment planning in this disease burdened region.

## 1 INTRODUCTION

Magnetic Resonance Imaging (MRI) is one of the safest and most versatile biomedical imaging methods available. Higher field strength systems ( > 1.5 tesla) can produce increased signal-to-noise pushing voxel size as low as hundreds of micrometers [1]. However, this capability comes at a cost of at least $1M per tesla [2] making these high-field systems inaccessible to those in low-to-middle income countries (LMIC). The unequal distribution of MRI and other medical technologies throughout the world has been well documented [3]. Even when technology such as MRI is introduced into LMIC through donation or purchase, more than half typically falls into disrepair and more than a quarter may never even be operational [4]. This is due to the lack of spare parts, consumables, trained technical staff, or reliable power in addition to the high initial cost of acquisition [5]. A sustainable solution is necessary if diagnostic imaging is to be a useful tool for clinical practice in LMIC.

The term “sustainability” has been used in many different contexts, even as it relates to medical care. Following a review of the uses of “sustainability” in medical research in which the authors attempted to aggregate the most common definitions used, this work most closely matches the category of “Continued program activities” [6]. Here we invoke “sustainability” in the most literal sense—we need technology that can operate in LMIC in the long term.

A condition representing high clinical need in LMIC is childhood hydrocephalus. Globally there are an estimated 400,000 new cases of pediatric hydrocephalus per year with over 90% in LMIC [7]. In sub-Saharan Africa where post-infectious hydrocephalus is most common, there are approximately 180,000 new cases per year [8]. These infants require treatment to survive. In Hydrocephalus there is a buildup of cerebrospinal fluid around the brain and within the ventricles, creating excess intracranial pressure. It is treated with either ventriculoperitoneal shunting [9, 10] or endoscopic third ventriculostomy (with or without choroid plexus cauterization, ETV-CPC) [11, 12]. For treatment planning, computed tomography is often employed in LMIC since it provides good spatial resolution and contrast between brain and CSF, and is less costly than high-field MRI, however the ionizing radiation associated with this technology has been shown to be a high risk especially for infants [13]. Ultrasound can be a useful tool, but only before the first year of life when the skull begins to fuse [14]. Fortunately, the imaging needs for hydrocephalus treatment planning are relatively straightforward—clinicians must visually separate CSF from brain with enough confidence to plan surgery. While typical high-field images can provide sub-millimeter voxel spacing, we have suggested that considerably larger voxels (3 × 3 × 10 mm^3^) could be sufficient for treatment planning [15] opening the door for the use of low-field MRI technology.

There has been recently renewed interest in clinical low-field MRI as an affordable and portable alternative to high-field imaging. Permanent magnet systems with main field strengths of 50 milli-tesla [16], 80 milli-tesla [17], and 64 milli-tesla [18] have all shown capability for clinically relevant brain imaging at varying levels of portability and cost. The system described by O’Reilley et al. (2020) has been made available in an open source forum for an estimated cost of 10,000 euros (https://www.opensourceimaging.org/project/halbach-array-magnet-for-in-vivo-imaging/). Coil-based low-field MRI offers another potential route to sustainable clinical imaging at even lower field strength. Work by Ref. [19] has shown impressive images of brain using a large Helmoltz coil design (220 cm diameter) with field strength of 6.5 milli-tesla. Although this system produces quality human brain images with a coil-based low-field MRI, it is not portable and consumes more than 4 kW of energy to generate the main, static field. Previous work from our group has described a prepolarized low-field MRI (PMRI) specifically designed to assist with treatment planning for infant hydrocephalus in Uganda [15]. Details of this specific magnet design are discussed in Ref. [15] and in further general detail in Refs [20–22].

While the work in Ref. [15] described the magnet design and testing for the PMRI system, the present work focuses on the radio frequency chain (RF chain) of electronics required to operate the PMRI system. The motivation behind this work was to design a rugged and low power option for sustainable operation in low resource countries like Uganda and provide documentation that can serve as a basic tutorial on how to implement the RF system. The RF chain is powered using two 12 volt tractor-style lead-acid batteries. We take advantage of the relatively low operating frequency of 180 kHz to allow components in our RF chain to remain unmatched to 50 ohm impedance. This allows the transmit (Tx) coil to be tuned to a minimum impedance, maximizing the current delivered to the coil. In addition, we employ commonly available animal enclosure fencing (chicken wire) as a Faraday shield and compare the effectiveness over more traditional shielding options. Finally, we show imaging capability using basic geometrical phantoms filled with water.

## 2 MATERIALS AND METHODS

**FIGURE 1 |:**
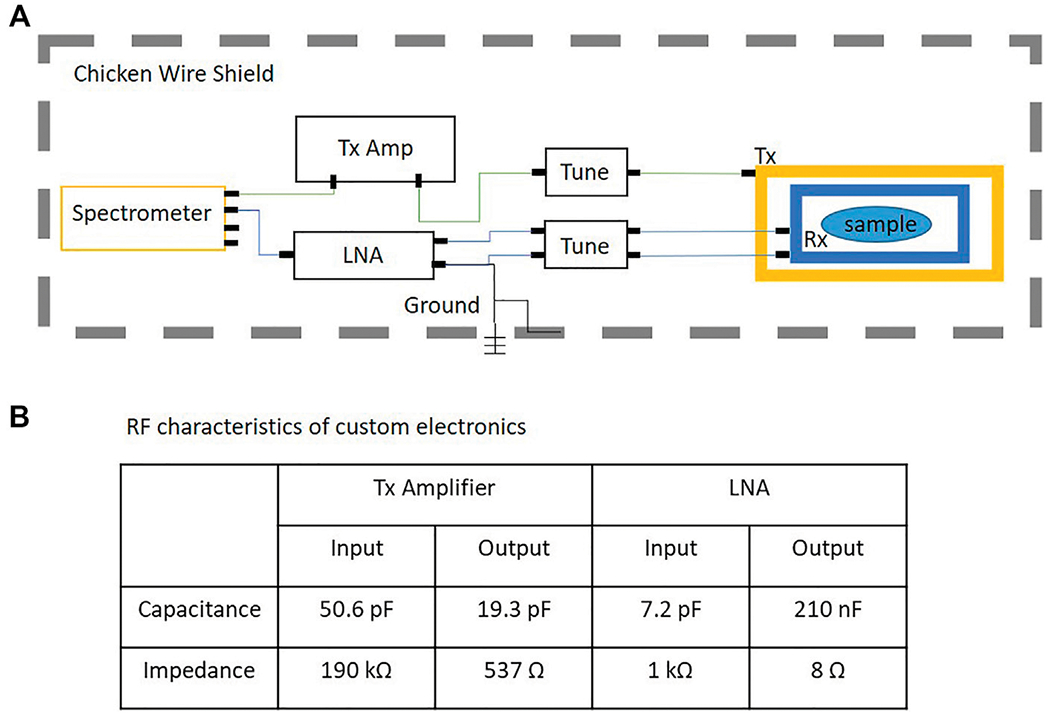
**(A)** Block diagram of the RF chain in the PMRI system; **(B)** RF characteristics of the Tx amplifier and LNA.

The Drive-L spectrometer developed by PureDevices was used for our PMRI system. Figure 1A shows the block diagram of the RF chain which includes a Transmit (Tx) amplifier, a Tx coil, a tuning network for the Tx coil, a receive coil (Rx), a tuning network for the Rx coil, a low noise amplifier for the Rx coil (LNA), and a shield surrounding the system. All analog-to-digital conversion takes place within the spectrometer.Figure 1B shows the RF characteristics of the Tx amplifier and LNA. The main magnet in this system generates a center field of 4.23 milli-tesla which corresponds to a Larmor frequency of 180.1 kHz.

### 2.1 Transmit and Receive Coils

Two separate RF coils were used for transmit and receive (Tx and Rx respectively). A saddle design was chosen for both since these coils are straightforward to build with copper wire and have a relatively uniform field. Table 1 shows the parameters of the two coils used. An initial calibration step is necessary to ensure the Tx and Rx coil are decoupled. For a two-coil system, decoupling can be achieved by rotating the coils so that their field directions are orthogonal to each other. This was done by connecting the Rx coil to an oscilloscope and passing a 180 kHz signal through the Tx coil at 5 *mV_pp_* with a function generator. When the coils are strongly coupled the signal amplitude will approach 5 *mV_pp_* as measured by the oscilloscope. Decoupling is achieved by rotating the Rx coil until a signal amplitude less than a microvolt is visible on the oscilloscope.

Litz wire has been suggested to be a more efficient wire for low frequency RF signals since it reduces the loss in current density along the wire produced by the skin effect. It has also has been shown to be useful in low field MRI applications [23]. In a circular conductor, current density is pushed toward the outside edge as frequency increases due to the changing internal magnetic fields caused by the alternating signal. This increases the effective resistance of the conductor. The optimal wire diameter should be twice the skin-depth of the wire material at the frequency of operation. A smaller wire also has increasingly small current carrying capacity, but if many small wires are soldered in parallel, the current can be split between them reducing the impedance. The Litz wire used in our PMRI system has 100 strands of AWG 36 wire.

### 2.2 Tuning Circuits

**FIGURE 2 |:**
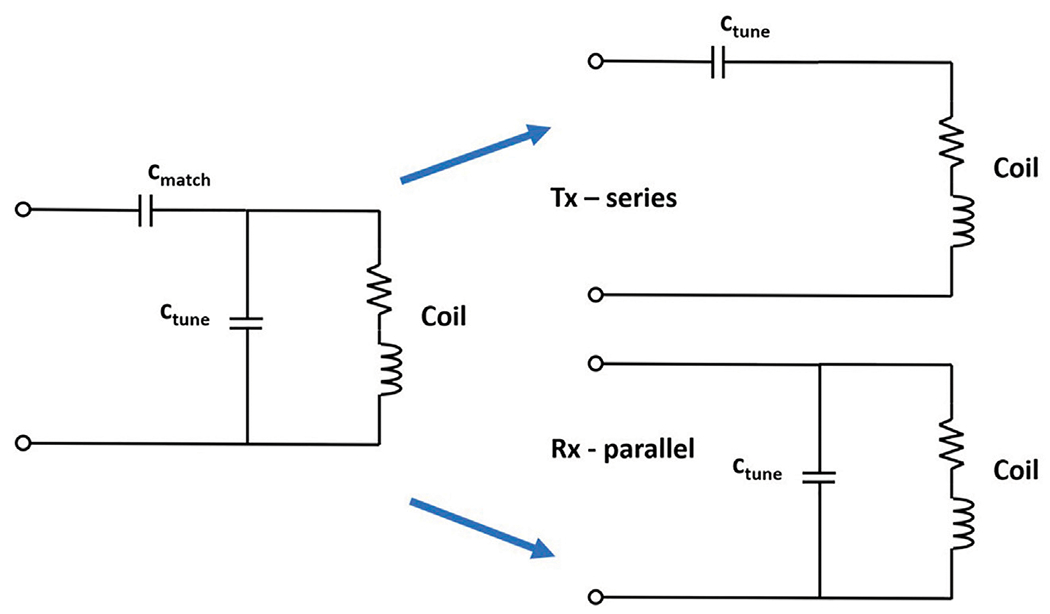
RCL circuit. Typical tuning of the RF coil uses a tuning capacitor in parallel (*c_tune_*) and a matching capacitor in series (*C_match_*). In the low-field design, Tx can be tuned in series, providing minimum impedance, and Rx can be tuned in parallel, providing a higher Q.

Both the Tx and Rx coil can be modeled as a resonant resistor-capacitor-inductor (RCL) circuit. For PMRI, the coil should be resonant at the Larmor frequency in order to detect the small signals (*μV*) resulting from low static magnetic field. A tuning capacitor is added in parallel with the coil to shift the natural resonant frequency to the Larmor frequency, as shown in Figure 2. A matching capacitor is typically added in series to match the impedance of the coil to 50 ohms. Addition of this capacitor alters the tuned frequency and one must use an iterative process to tune and match. In the low-field system at 180 kHz, matching is not a requirement and it may be desirable to have minimum impedance in the Tx coil while maintaining a higher impedance in the Rx coil.

It is a well-established principle in RF engineering that an impedance mismatch between input and output ports of two connected RF components leads to reduction in power transmission efficiency. It has also been shown that matching impedance of source and load between each component in a chain of RF electronics provides the most optimal power transfer efficiency [24]. For historical reasons, most RF electronic components and cables are impedance matched to 50 ohms.

There are specialized cases where ports and cables are not matched to 50 ohms. Test equipment that is sensitive to small voltages may have high impedance (100 Mohm or more) so that voltage drops preferentially on the input port of the measurement device. In some audio/video applications 75 ohm transmission cables and impedance matching is used. For most mid- to short-wave frequency applications, 50 ohm matching is the standard.

The power attenuation from impedance mismatch comes from the reflection of the transmitted wave at the input back to the source or to the surrounding space. When the reflected wave combines with the incident wave to create standing waves, peaks and nodes occur along the transmission line. The measurement of this phenomenon is often called the Standing Wave Ratio (SWR) [25].

The effect of SWR on efficiency is dependent on transmission frequency and the length of the transmission cable [25]. Significant reflections can be avoided if the cable is much less than one-quarter of the frequency wavelength. This rule of thumb makes impedance mismatch possible at low RF frequencies without sacrificing much efficiency. For example, at our typical operating frequency of 180 kHz, the wavelength is around 1,600 m. Since the cables used in the PMRI system rarely exceed 1 m they are not considered “long,” and therefore energy loss at the load due to reflection at this frequency is virtually non-existent. This means an impedance mismatch will not lead to significant power inefficiencies.

Relaxation of the impedance matching principle provides more flexibility in designing electronics that are cost-effective and low power. For example, if the transmit amplifier is not driving 50 ohms, it could be low voltage and still deliver a useful amount of current. Since the impedance of a series RCL circuit has a frequency dependent minimum at resonance, a series capacitor is used to tune the Tx coil, as shown in Figure 2. Conversely, the impedance of a parallel RCL circuit has a frequency dependent maximum at resonance, so the parallel resonant circuit is used to tune the Rx coil as shown in Figure 2. By creating a series resonant RCL circuit at the desired frequency, impedance can be shifted to a minimum approaching the DC resistance of the Tx coil. Inefficiencies in passive electronic components and residual skin effect in the wire of the Tx coil make the impedance higher than DC resistance, but we were able to tune our coil to 180 kHz at an impedance of 5 ohms, providing a > 5X current increase per signal voltage over the 50 ohm, impedance matched case.

In our low-field system, a 1 milli-second pulse with a 1 A_pp_ current delivers the desired *π*/2 flip angle. For a coil tuned to 180 kHz with an impedance of 5 ohms, this requires a 5 V_pp_ signal, or 25 W, compared to the 50 ohm impedance matched case, which would require 25 V_pp_ and over 125 W. In addition to an increase in power, the required voltages and increased power dissipation does carry significant design implications for the Tx amplifier—namely number of standard op-amps required to drive the load and number and type of required power sources for the op-amps.

### 2.3 Transmit Amplifier

The Tx amplifier was designed to operate with a minimum impedance Tx coil. The key aspect in designing an RF amplifier is choice of the correct operational amplifier (op-amp). For the specific aims of this project the op-amp should meet the voltage and current requirements of the application, maintain signal integrity, be as readily available throughout the world as possible, be of the appropriate size to work without specialized equipment, and be affordable in low quantities. The circuit design should also be as simple as possible to allow for fewer components and low-tech construction or repair.

Texas Instruments (TI) op-amp OPA-549 was chosen to drive the voltage amplifier. The op-amp data sheet is available on the Texas Instruments website (www.ti.com) and the circuit design for the Tx amplifier can be found in the supplemental data. The op-amp is capable of 8 A continuous output and can be driven by up to ±30 volt. Open loop gain is linear at the frequency of intended use and noise voltage density is at a minimum of $70\mathrm{nV}/\sqrt{Hz}$.

Power supply requirements are important to consider in terms of heat dissipation and rail-to-rail voltage. Consider the case where one might want to drive a 50 ohm impedance matched RF coil at 180 kHz with an OPA-549 amplifier. If we want to drive 0.5 A of current we need a 50 V_pp_ swing (25 volts in each direction). The rail-to-rail voltage of a differential amplifier (i.e. the voltage limit in the positive and negative direction before the signal is clipped) is typically slightly less than the supply voltage, and is often specified in the op-amp documentation. This means, for a single OPA-549, we could supply this desired signal amplification with a ±30 volt power supply, but we would need to consider a good cooling strategy for the op-amp. We would also be utilizing a very small amount of the available current output for the maximum voltage supply.

Alternatively, we chose to build a bridged amplifier with two OPA-549 op-amps—one per supply pole. This allows us to divide the heat dissipation across two op-amps and reduces our need for cooling. It also maintains the current driving capability while essentially doubling the rail-to-rail voltage of the amplifier. This means that we could supply 60 volts to each side (roughly 120 volts rail-to-rail) and even drive the coil with 1 A of current. If more current is desired, op-amps can be added in series to each polar side of the design. This design meets the amplification needs of our system without requiring forced cooling strategies.

Since the Tx coil in our system was tuned to 5 ohms instead of 50 ohms the power supply strategy can be simplified to two 12 volt supplies. We can supply the OPA-549 with ±12 volt to produce a rail-to-rail of 20 V_pp_, or we can bridge two amplifiers and supply each with 12 volt and drive a little over 1 A of current without excessive heat dissipation. This method has a few other advantages. Many other electronic components in the system are driven with 12 volt power supplies, which means we can couple them all to two 12 volt linear power supplies, or power the entire RF chain using two 12 volt car or tractor batteries. This has many sustainability advantages when it comes to use in LMIC.

The Tx amplifier is capable of 8 A continuous (10 A peak) output with a rail-to-rail voltage of around 20 volts before clipping. The Tx coil was tuned to 5 ohm impedance which allows for a maximum of 4 A delivered to the coil. For the spin-echo imaging used in the PMRI system, minimum pulse widths of between 0.5 ms and 1 ms are typically used for the *π*/2 pulse with 1 A current. shorter pulse widths could be desirable, in which case supply voltage can be increased or the amplifier can be bridged to increase the rail-to-rail voltage.

### 2.4 Low Noise Amplifier

One of the most important components of the RF chain is the low noise amplifier (LNA). The LNA is used to amplify the small signal coming from the receive coil, which can be as low as *μV* for PMRI. A good LNA design will add the appropriate amount of gain over the bandwidth of interest without adding a significant amount of noise. Unfortunately, it is difficult to tune all of these parameters simultaneously. A small amount of noise is unavoidable, since these are active electronic devices constructed using transistors. For RF signals, transistor performance depends on source impedance, frequency, and amplifier gain [26]. In order to optimize noise performance, matching networks are often used to transform the source impedance from 50 ohms to the optimal impedance at the target frequency, often at the expense of gain. For most applications, the target source impedance is much more than 50 ohms and has been suggested to be closer to 1 kohm [26].

Placement of the LNA within the system can also be important. Long cables connecting the coil to the input of the LNA can attenuate signal, add thermal noise, and introduce other types of noise artifact such as interference from other systems or movement of wires. The LNA is often placed as close to the coil as possible, bearing in mind that the strong magnetic fields can induce unwanted currents through the Hall effect [27].

Lastly, in many receive chains there are other components after the LNA which could also contribute to the amount of noise added to signal after reception. This is often referred to as the noise figure (NF) which is a measure of the noise added to the signal by a circuit element. As the first electronic component in the chain, the NF of the LNA dominates that of the other components and so greatest care must be taken in its design [27]. For our low-field system, many of these design restrictions can be relaxed. For example, at 180 kHz a 1 m cable connecting the Rx coil to the LNA will add very little attenuation and minimal resistance. Lower frequency also reduces the likelihood of parasitic capacitance within the board layout, and so a more relaxed construction approach can be realized. Unfortunately, most out-of-the-box LNAs are usually optimized for higher frequency applications and can be quite expensive.

**FIGURE 3 |:**
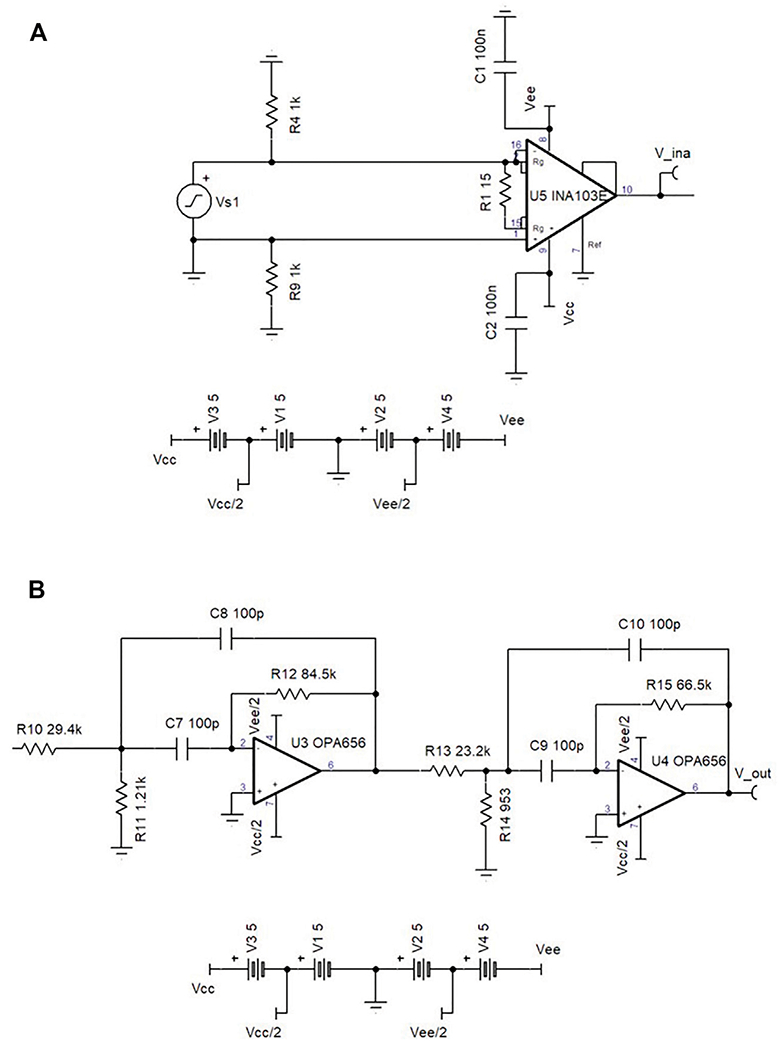
**(A)** The circuit diagram for the custom LNA design using TINA software from Texas Instruments. **(B)** The circuit diagram for the 4th order butterworth filter used at the output of the LNA.

We designed an LNA to meet the specifications outlined in Table 2. The actual build specifications are also listed for comparison. The circuit diagram of the LNA can be found in Figure 3A. The LNA is an instrumentation amplifier with a 60 kHz bandwidth Butterworth filter centered at 180 kHz on the output.

The choice of op-amp for the LNA is the most important aspect of the design architecture. A single-ended op-amp has a single input and output, both referenced to ground. This creates a simple architecture, but does not reject common mode signals arising from interference on signal lines. A differential amplifier has differential input and output. The output becomes the gain times the subtraction of the two input lines. This means any signal common to both lines will be removed from the output, which is useful in high external noise scenarios [28].

Instrumentation amplifiers have all the benefits of a differential amplifier with the added benefit of single-ended output and, often, lower noise [29]. An instrumentation amplifier op-amp (INA-103) from Texas Instruments (TI) was chosen for the LNA design. The instrumentation amplifier has three internal op-amps. The two input amplifiers act as differential input rejecting common mode at a common-mode rejection ratio (CMRR) of 100 dB. The third stage combines these signals and delivers a single ended output referenced to ground. Another useful feature is that gain is set by adding a single external resistor at pin 14, while the internal feedback resistors are laser-trimmed (3 *k*Ω ± 0.1%) which provides excellent gain balance between the internal differential stages. INA-103 has excellent noise performance for source impedance below 10 *k*Ω. In addition, the amplifier acts as low-pass filter for frequencies above 1 MHz. This is useful for eliminating high frequency noise, but with an operating frequency at 180 kHz we chose to add a bandpass filter as well.

An active fourth-order Butterworth bandpass filter with two stages was designed and added to the output of the LNA, as can be seen in the circuit diagram in Figure 3B. At high frequencies, passive filters are often designed using reactive elements such as inductors and capacitors. At low frequency, the inductor values can become too large to be practical, adding unwanted time delays in the circuit, and so active circuits are preferred. The bandpass filter was constructed with two OPA-656 op-amps. It has a unity gain bandwidth of 500 MHz, $7nV/\sqrt{Hz}$ input noise voltage, and a 60 kHz bandwidth around the 3 dB attenuation points. Table 3 shows the specifications of each stage in the bandpass filter. While the 500 MHz bandwidth was not an important aspect of the design consideration and is considerably wider than required, unity gain stability in the bandpass filter avoids amplification of noise added in the filter stage.

**FIGURE 4 |:**
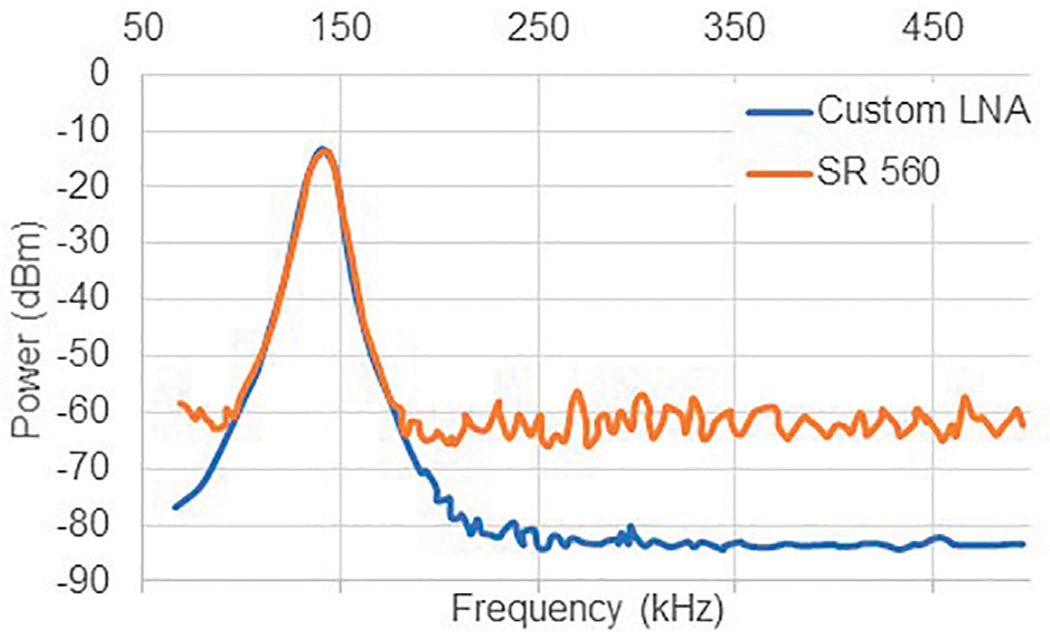
A comparison of the noise floor and signal level between the custom LNA and the SR560. The custom LNA shows a 20 dB improvement in noise floor over the SR560. Data points were extracted and plotted from an analog spectrum analyzer.

This amplifier was designed to replace a high end LNA made by Stanford Research Systems (SR560). While the SR560 is an excellent LNA, it has a high NF (24 dB) at the frequency and source impedance of the PMRI application. An experiment was designed to compare the LNA designed in this work to the SR560. A 1 mV_rms_ signal was sent to the Tx coil, received by the Rx coil tuned at 180 kHz, and amplified with both the LNA and SR560. The output of the LNA or the SR560 was measured by a spectrum analyzer and the noise floor around the signal was recorded. Figure 4 shows the comparison. The LNA shows an improvement of 24 dBm over the SR560 in this application. This is an illustration of how simple, purpose built electronics can outperform bench-top solutions at a fraction of the cost. The LNA developed in this work costs around $100, while the SR560 costs around $5,000. It should be noted that the SR560 was not designed to be a low noise amplifier at frequencies or source impedances as low as this application requires and we should not expect high performance for our application. This example is intended to illustrate the cost-savings that can be achieved through simple, custom built designs.

The LNA is powered by two 12 volt linear power supplies or two 12 volt batteries. An internal circuit is used to step down the power voltage to the filter op-amps to ±5 volt.

### 2.5 Shielding

The last component of the RF chain to discuss is the shield for the system. For high-field MRI systems, a well shielded room is an expensive and necessary component in order to achieve the high SNR these systems are designed to exhibit. Although shielding for low-field systems can be much simpler and more affordable, it is still an important component of maximizing available SNR that must be addressed.

The first consideration is the size of the shield. An excellent solution for reducing cost with good shielding effect is to build an aluminum Faraday cage around the system alone, leaving the room unshielded. This also allows for portability of the system, an important component to sustainable design. This design was used in [16] to great effect, producing good quality low-field brain images *in vivo.* One potential risk with this method is that one side of the shield must be left open for the patient to enter the machine. The patient also acts as an antenna as he or she couples with the RF receive coil. In [16], a conductive aluminum cloth grounded to the shield was used to cover most of the patient’s body, which reduced noise by a factor of 10. The downside of such a shield is that the conductive cloth is quite expensive and could be hard to acquire in the developing world. The small shielded enclosure is also hard to see inside and may not be ergonomically designed for infant imaging.

Another option is to construct a room sized enclosure, large enough to seat at least two adults (parent and technician) and image an infant. One potential advantage of such a system is that, with low-field MRI, ferric material that is relatively far from the system will not distort the magnetic field or turn into a dangerous projectile. This means there is the potential to use ferric metal as the shield material, such as an alloy of steel. In our experience, steel is typically less expensive and more readily available (especially in LMIC) than aluminum or copper and would be a preferred option for sustainability reasons. A drawback of this design is that it is not portable, unless built around a truck-bed or in a trailer, and the larger size could incur more cost. The use of inexpensive steel materials may be able to offset the cost of larger size.

Another fortuitous aspect of shielding at low frequency, such as 180 kHz, is that it is much easier to construct an ergonomic enclosure with a focus on patient comfort without embedding the shield in the walls of the room. This can be achieved by using perforated material as a shield. If designed properly, perforated material can be as effective as solid material and it allows for good airflow and reduces claustrophobia.

In electromagnetic interference (EMI) shielding theory, there are three main aspects of the shield design that should be considered as it relates to the target frequencies to be shielded: 1) the shield material, 2) the thickness of the shield, and 3) the size of any gaps in the shield. In some applications, shields are designed to be effective for electric and magnetic fields, however for the PMRI system we focused on shielding electric fields. Electrical interference interacts with the shield by reflection, absorption, and transmission [30].

**FIGURE 5 |:**
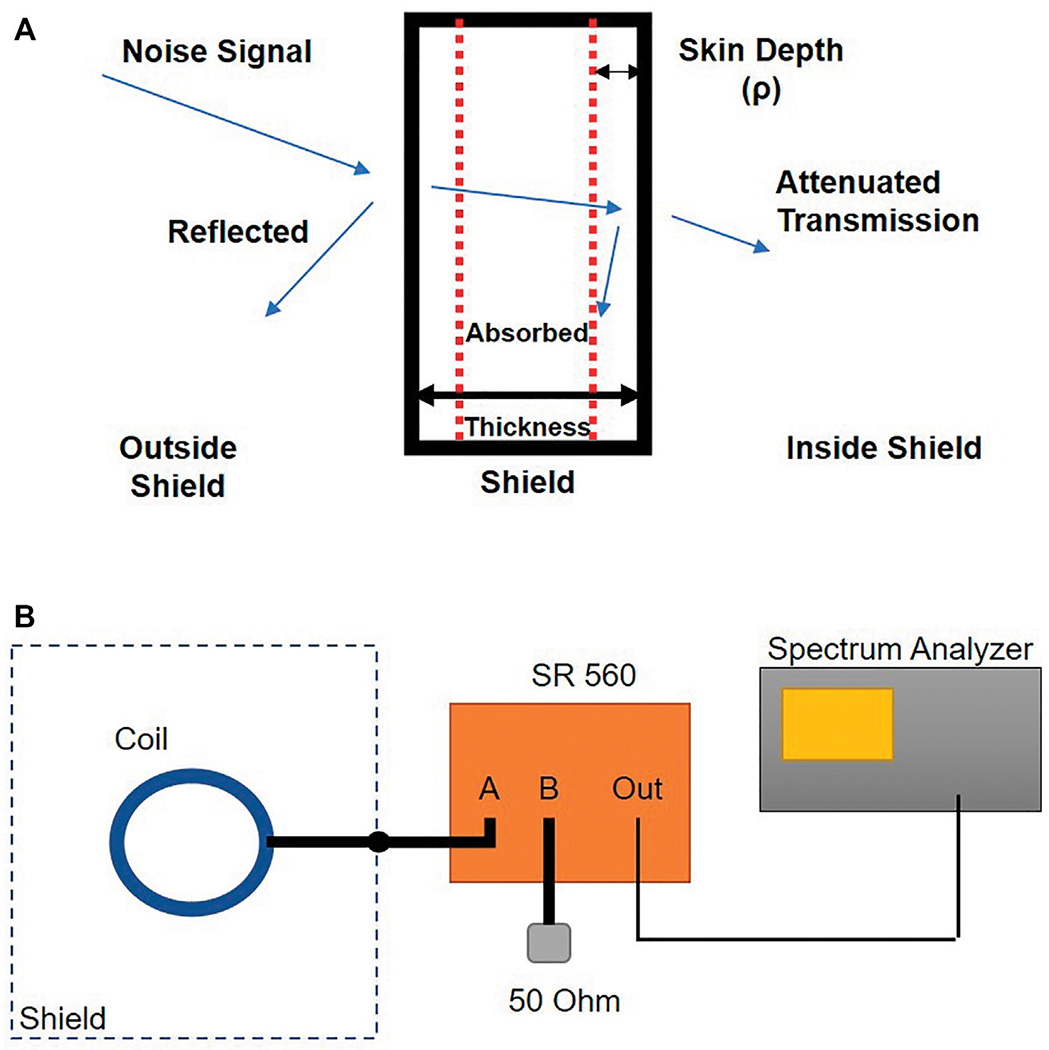
(**A)** Cross-section of an EMI shield. Interference signals are shielded by external reflection or absorption of RF energy. Depending on the efficiency of the shield, an attenuated interference signal is re-transmitted into the shielded space. **(B)** Shield test setup. The coil was placed inside each shield with coil cable grounded to the shield and connected to input A of the SR 560 low noise amplifier. A 50 ohm resistor was connected to input B of the amplifier. Output was measured by a spectrum analyzer.

Figure 5 shows a cross-section of an EMI shield interacting with a noise signal outside the shield. Depending on the shield material and frequency of the noise signal, some of the incident wave will be reflected and some will be absorbed. The amount of the incident wave absorbed depends on the shield material and frequency of the wave, but also on the thickness of the shield material. To understand this, we need to look at how AC current is carried in conductors.

A DC current uses the entire cross-section of a conductor to move charge. As frequency increases in an AC current, a back-EMF is generated the by the alternating charge through increasing magnetic field at the center of the conductor. This increased magnetic field looks like higher and higher impedance to the current, and charge density is pushed toward the edge of the conductor. This effect also increases the resistance of the conductor as frequency increases. This phenomenon is called the skin-effect. The cross-sectional area in which more than 37% of the charge is being carried is called the skin-depth, as measured from the surface of the conductor. The equation for skin depth is [31].

(1) $$\delta =\sqrt{\frac{2}{\omega \mu_{o}\sigma }}$$

where *ω* is the angular frequency of the AC signal, *μ*_*o*_ is the permeability of a vacuum, and *σ* is the conductivity of the material. From Eq. 1 we see that as frequency increases skin depth decreases. This is important for the design of the thickness of the shield because it will determine the upper limit frequency that can be effectively absorbed by the shield. If the shield thickness approaches the skin depth of the target frequency, the shield will not behave as an effective conductor and most of the energy that is not reflected will be re-transmitted into the enclosure. As a rule of thumb, the shield should be more than five times the thickness of the skin depth [30]. Comparisons between frequency, material, and thickness can be made using the shielding effectiveness nomograms printed in Ref. [30].

For the PMRI signal, we want to shield frequencies around 180 kHz. Since the Low Noise Amplifier has a 60 kHz bandwidth filter on the output, we at least must shield frequencies that will be passed by this filter. In practice, this is an easy case to design for. If we take the highest important frequency to be 240 kHz (a full filter bandwidth above center), the wavelength of this frequency is over 1,200 m. This means that, although the best case scenario shield is a solid box with no gaps, we should be able to use a perforated material to allow for better airflow. When choosing material, an obvious choice is aluminum or copper. The skin depth of 240 kHz in these materials is *δ*_*Al*_ = 0.2 *mm* and *δ*_*Cu*_ = 0.15 *mm*. Since the PMRI system has such a low field and we plan to build an enclosure that is much larger than the 5 Gauss line, we might also consider steel with a skin depth of *δ*_*Steel*_ = 1 *mm*.

Three small enclosures were constructed to test the best case to worst case scenario for shielding for comparison. Each box was 1 × 1 × 1 ft^3^ in size. Box 1 was made of 10 mm thick solid aluminum, box 2 was made of 10 mm thick perforated aluminum with 10 mm diameter perforations, and box 3 was made of steel chicken wire with a 12.7 mm square grid pattern.

The tests were performed using a 50 ohm impedance matched single-ended saddle coil resonating at 180 kHz. Coil location in the room was consistent across tests. The coil was placed in each shield with the coaxial cable grounded to the shield. The SR 560 low noise amplifier made by Stanford Research Systems was used to amplify the signal with a gain of 100x. The noise spectrum was analyzed with a spectrum analyzer. Each shield case was compared to the baseline noise of a 50 ohm resistor connected to port B of the amplifier. A fourth scenario was tested without any shield to establish the maximum noise picked up by the coil in the environment.

It can be seen from Table 4 that perforated and solid aluminum are the best performers, at least to the noise floor of the spectrum analyzer, since they both match the noise power of the 50 ohm resistor. Interestingly, the chicken wire shows a 10 dBm improvement over no shield, and only lags the aluminum options by 5 dBm. If we consider patient comfort, then the perforated aluminum option is better than the solid aluminum since it allows for air flow. The cost of the perforated aluminum is roughly 10 times that of the chicken wire and so chicken wire could be an excellent option for a shielded enclosure on a sustainable system targeted to the developing world.

**FIGURE 6 |:**
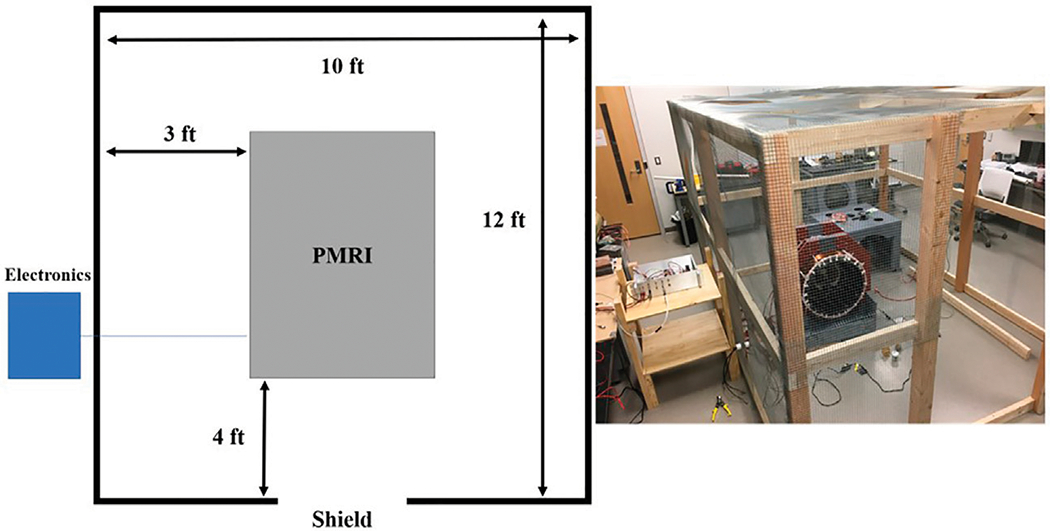
Footprint of the shield enclosure (left) with image of constructed shield (right). The shield was constructed using 2 × 4 s and chicken wire.

The shielded enclosure for the PMRI system was designed to be large enough for two adults to freely move inside the enclosure during imaging. Figure 6 shows the footprint of the shield enclosure. There is at least 1 m between the PMRI system and the shield to allow for a technician to access any part of the system with ample space. The enclosure is 188 cm tall—large enough to accommodate a tall adult. The shield frame is made from common lumber used for framing small structures (in this case, 2 × 4 inch lumber) and brass screws. The shield material is chicken wire as described above. The total material cost of the shield was $300 USD, a 10X cost reduction over a shielded enclosure with perforated aluminum.

## 3 RESULTS

**FIGURE 7 |:**
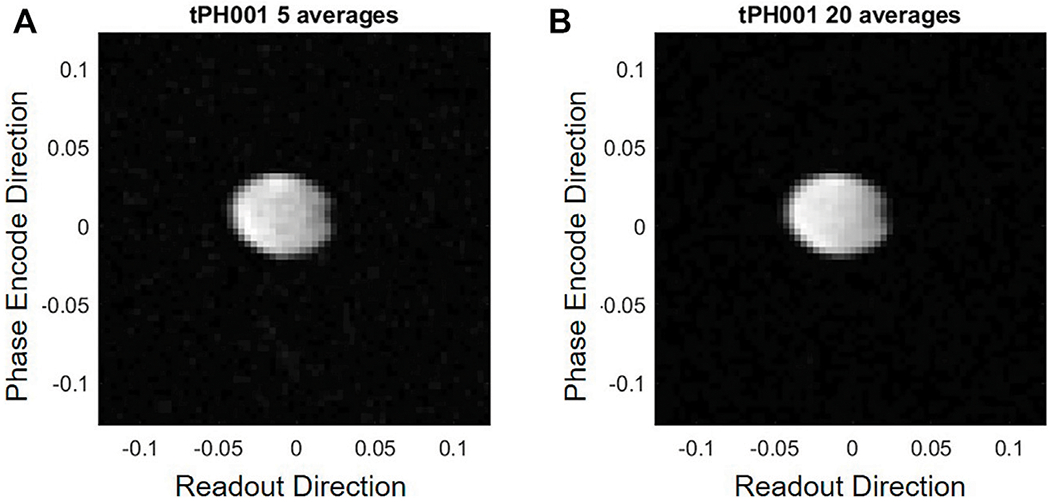
Comparison between 5 averages (SNR = 23) **(A)** and an 20 averages (SNR = 39) **(B)** for a turbo spin echo sequence of image the cylindrical water phantom.

**FIGURE 8 |:**
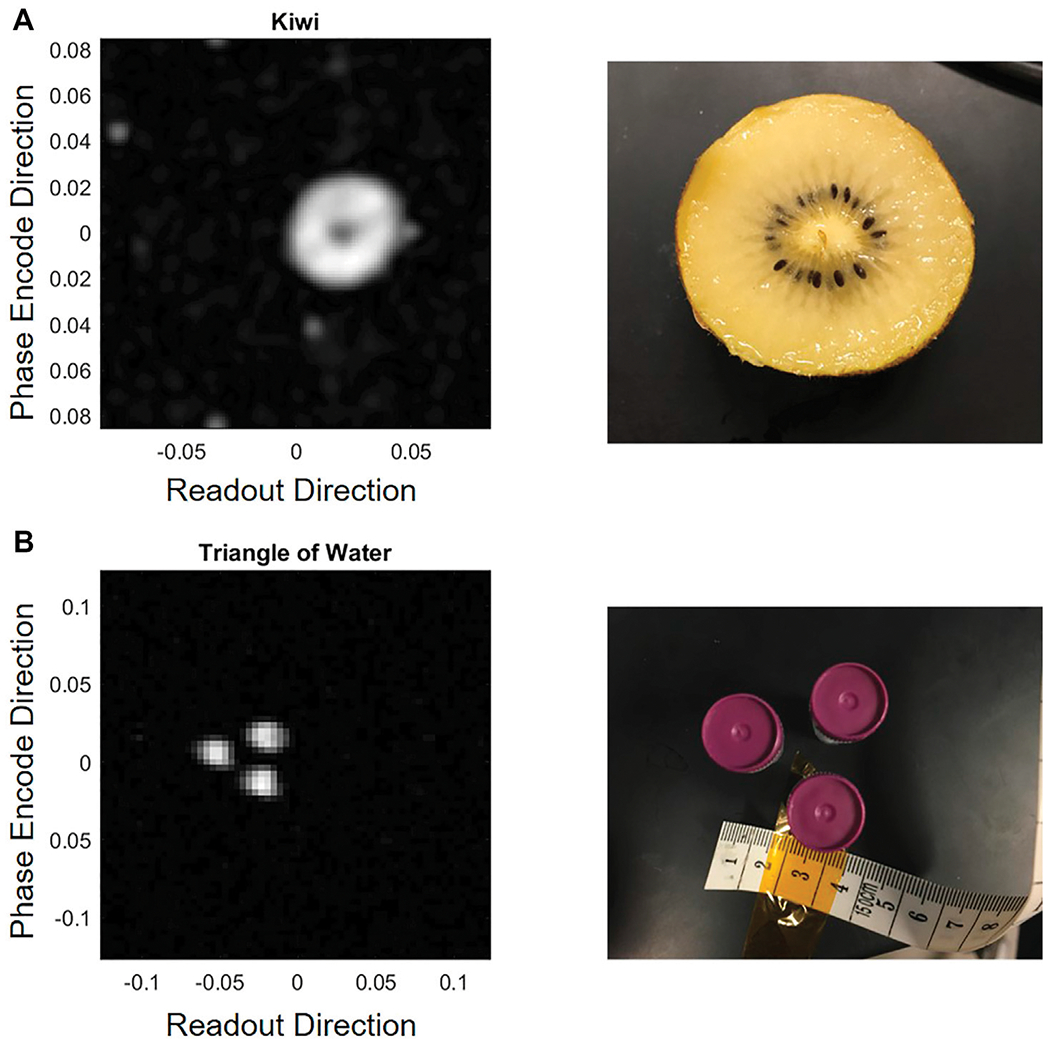
Images of a kiwi **(A)** and three small bottles of water arranged in a triangle **(B)**.

The PMRI system can achieve an SNR of 10 for the cylindrical water phantom with a 64 × 64 resolution FOV of 25 cm^2^ and using 5 averages with 50 mT Bp field (20 Amp, 75 volt battery power). Figure 7 shows the cylindrical water phantom measured with 5 averages (A) and 20 averages (B). Although the SNR roughly doubles by using 20 averages instead of 5 averages, the imaging time increases by 4. Figure 8 shows images of a kiwi (A) and three small water bottles arranged in a triangle (B).

Imaging experiments were performed in order to show the capability of the designed electronics. To determine SNR capability, a phantom was constructed using a plastic cylinder 5 cm long and 6.5 cm in diameter and filled with distilled water. This phantom is ideal for testing since it fills most of the Rx coil described in this work and is not long in the *z* direction. This helps to minimize inhomogeneity effects caused by unwanted Bm gradients in the *z* direction but still allows for a significant amount of signal for imaging in the x-y plane. A turbo spin-echo (TSE) sequence was used with a turbo factor of 16 on a 64 × 64 matrix image. The turbo factor defines the number of TR blocks that will be used in the TSE sequence. A turbo factor of 16 for a 64 × 64 image means there will be 4 TR blocks. The Bp was on for 4 s prior to imaging and there was a 550 ms wait time between turning off the Bp and starting the imaging sequence. This wait time causes a 27% loss in SNR due to T1 decay. The receiver bandwidth was 50 Hz per pixel with a *π*/2 pulse of 1 ms. The effective echo time was 80 ms.

Although this basic phantom is useful for troubleshooting, more complex phantoms were also imaged. To show capability of imaging a phantom with more complex internal structure, a kiwi was imaged using a TSE sequence with a turbo factor of 32 and with echo time of 50 ms, and Bp duration of 3 s with a wait time after Bp before imaging of 450 ms. The Bp was powered to 50 mT by batteries. Image resolution was set to 32 × 32 with a 17 cm^2^ square FOV. A 75 Hz/pixel bandwidth was used with a 1 ms *π*/2 pulse. The image of the kiwi can be found in Figure 8.

In addition, three small water-filled tubes arranged in a triangle were also imaged as can be seen in Figure 8. The water tubes were each 5 cm long with a diameter of 1 cm. The triangle was imaged with the 50 mT Bp field and 20 averages using the same imaging sequence as the cylindrical phantom.

## 4 DISCUSSION

Components of the RF chained designed in this work were successfully paired without the need for 50 ohm impedance matching. Not only did this reduce the complexity of the circuit design and construction, it more importantly reduced the energy required to power these circuits. Our goal was to design an electronic system that could be paired with the magnet described in [15] and which had the potential for use in hydrocephalus imaging. At the time of this writing, our colleagues at the Mbararra University of Science and Technology in Mbararra, Uganda have reproduced the coil-based PMRI system and electronics.

Despite this success, there is a drawback to the un-matched RF chain. When matching to 50 ohms, a component such as a coil can be replaced or changed without altering the experimental parameters or power efficiency of the RF chain. When the RF chain is unmatched, adding of a new component with different RF characteristics from the old component will require adjustment and calibration. For example, switching a Tx or Rx coil in the system will require recalibration of Rx pulse width and amplitude to achieve the optimal flip angle. This could be a significant drawback considering SNR is improved when coil size closely matches the object it is measuring. Further investigation should explore whether the power and simplicity gained in the unmatched case prove beneficial even in systems that are permanent magnet based. These systems already have a power advantage over coil based systems and an SNR advantage since they tend to produce larger static fields.

The imaging experiments in Figures 7, 8 demonstrate the capability of this affordable and low-power MRI system. The water phantoms show that with proper shielding and common post-processing techniques even low-voltage signals have enough SNR to accurately image collections of water. The image of the kiwi in Figure 8A further illustrates the ability of this system to show contrast between regions of the kiwi with high water content and low water content. Other details, such as seeds, would be hard to see at this resolution. Figure 8B shows that our system accurately captures the spacing and size of the water bottles, suggesting the possibility of 4 mm resolution with a 64 × 64 image.

In the context of hydrocephalus treatment planning, these are promising results, where we are most interested in accurately imaging large fluid collections and distinguishing them from brain. The water triangle phantom in Figure 8B demonstrates resolution accuracy that is on the verge of our target resolution for hydrocephalus imaging, as discussed in [15]. While the Rx coil used in this study is too small for infant head imaging, the rest of the system is appropriately sized and future work could likely provide similar results with a larger coil.

Another important aspect of this system is that these images were generated using very affordable and common electronic components such as tractor batteries, hand soldered electronics, and chicken wire. We demonstrate that low-field MRI opens the door to system design that does not require the strict tolerancing and high-end fabrication techniques common to the field of MRI technology. In this way, systems that can be built and maintained in low-resource regions of the world have the potential to aid in clinical decision making as with the example of infant hydrocephalus treatment planning in countries like Uganda. To that end, the MRI system employing both the PMRI coil described in [15] and accompanying electronics are actively being reproduced by our collaborators at the Mbarara University of Science and Technology in Mbarara, Uganda.

## 5 CONCLUSION

RF chain design is an important aspect of an MRI system, especially at low magnetic field where SNR is also typically low. Despite this, simple electronics and affordable shield materials, such as chicken wire, can provide high enough SNR to visualize internal structures at a low resolution. By leaving RF chain components unmatched to 50 ohms, we further simply the design and reduce RF power requirements during transmit. The design strategy for the RF chain compliments our efforts to develop a sustainable low-field MRI for use in the developing world.

While advanced low-field MRI solutions are becoming available that offer exciting clinical possibilities for a variety of diseases, we have demonstrated the capability of a low-powered RF chain for our portable PMRI system which was designed for treatment planning of hydrocephalus in low-resource settings. The power efficiency and simplicity, and low cost of the RF chain design offer the potential for a system that can be sustained in LMIC with primarily local resources. Further development and adoption of this technology will allow for access to diagnostics in rural populations where there are few current options that could have substantial clinical impact throughout the world.

## Supplementary Material

Supplement **Supplementary Figure S1 |** The circuit diagram for the Transmit amplifier.

## Figures and Tables

**TABLE 1 |:** RF coil properties.

Coil	Wire Type	Diameter (cm)	Length (cm)	Turns	Impedance (ohms) @ 180 kHz
Rx	100/36 Litz	10	12	10	1,508
Tx	100/36 Litz	24	25	20	10

**TABLE 2 |:** LNA Design Spec vs. Actual.

Spec	Gain (dB)	CMRR (dB)	NF	Settling Time (μs)	Center Frequency (kHz)	Bandpass BW (kHz)
Design	50	100	1	10	180	60
Actual	50	100	1.2	1.47	180	60

**TABLE 3 |:** Bandpass filter specifications.

Spec	First Stage	Second Stage
Center Freq (kHz)	160	202
Min. GBW (MHz)	68.3	86.6
Stage Gain (V/V)	1	1
Stage Q-factor	4.272	4.272
Topology	Multi-feedback	Multi-feedback

**TABLE 4 |:** Shield effectiveness comparison.

Condition	Noise Power at 180 kHz (dBm)
No Shield	−76.6
50 Ω	−89.6
Chicken Wire	−85.1
Perforated Al	−89.6
Solid Al	−89.6

## Data Availability

The raw data supporting the conclusion of this article will be made available by the authors, without undue reservation.
