# Supplementary figures and images for: An Unmatched Radio Frequency Chain for Low-Field Magnetic Resonance Imaging
Source: Front Phys. Author manuscript; Available in PMC 2022 Oct 6. (PMC9536774; doi:10.3389/fphy.2021.727536)

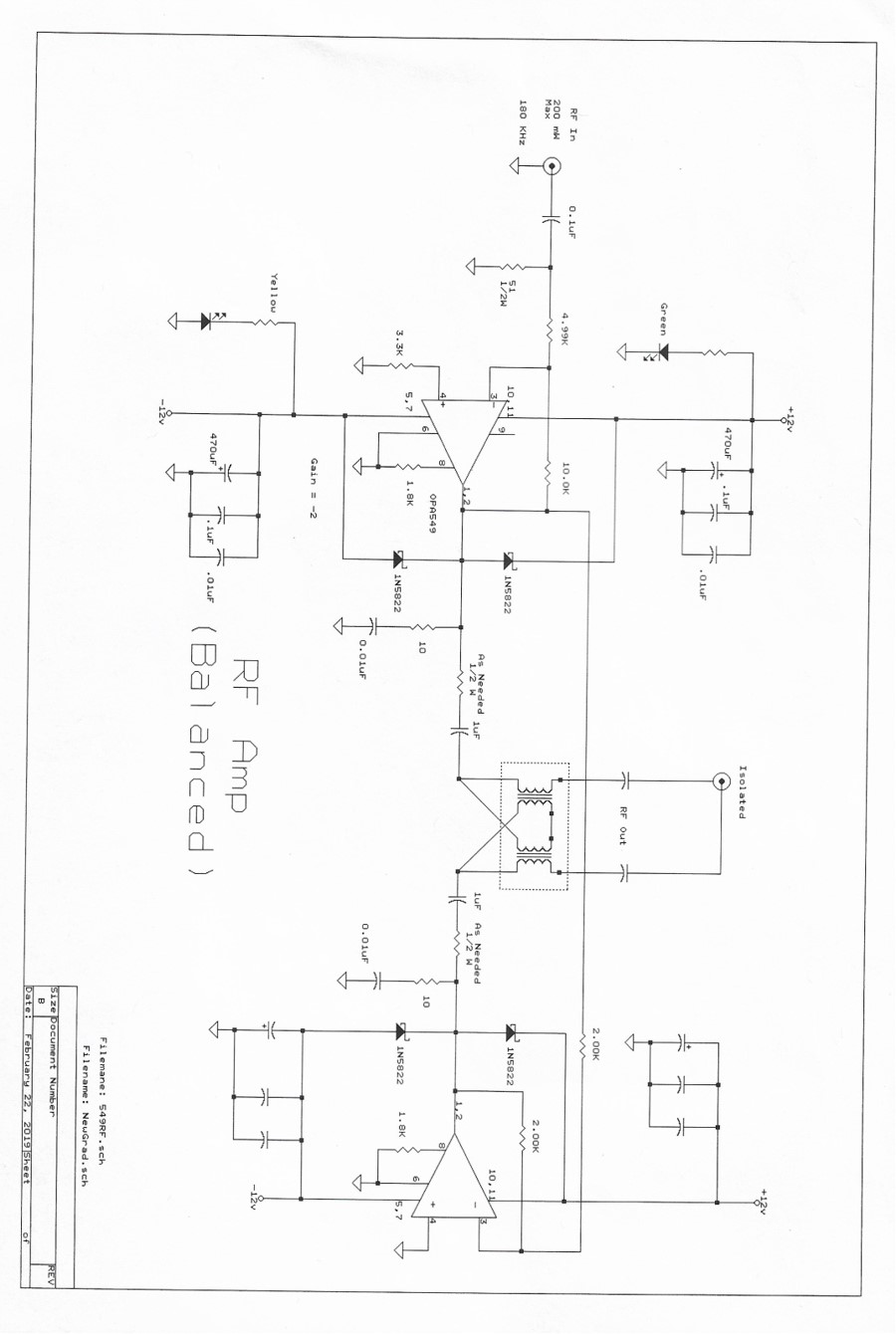

Supplement: Supplement — ary Figure S1 | The circuit diagram for the Transmit amplifier. [file NIHMS1802783-supplement-Supplement.jpeg]
